# Pulsed electromagnetic fields for the treatment of tibial delayed unions and nonunions. A prospective clinical study and review of the literature

**DOI:** 10.1186/1749-799X-7-24

**Published:** 2012-06-08

**Authors:** Aggelos Assiotis, Nick P Sachinis, Byron E Chalidis

**Affiliations:** 1 Charring Cross Hospital, Orthopaedic Department, London, UK; 2 Interbalkan Medical Center, 10 Asklipiou Str, Pilaia, Thessaloniki, 57001, Greece

**Keywords:** PEMF, Tibia, Fracture, Nonunion, Delayed union

## Abstract

**Background:**

Pulsed electromagnetic fields (PEMF) stimulation for the treatment of bone nonunion or delayed union have been in use for several years, but on a limited basis. The aim of this study was to assess the overall efficacy of the method in tibial delayed unions and nonunions and identify factors that could affect the final outcome.

**Methods:**

We prospectively reviewed 44 patients (27 men) with a mean age of 49.6 ± 18.4 years that received PEMF therapy due to tibial shaft delayed union or nonunion. In all cases, fracture gap was less than 1 cm and infection or soft tissue defects were absent.

**Results:**

Fracture union was confirmed in 34 cases (77.3%). No relationship was found between union rate and age (p = 0.819), fracture side (left or right) (p = 0.734), fracture type (simple or comminuted, open or closed) (p = 0.111), smoking (p = 0.245), diabetes (p = 0.68) and initial treatment method applied (plates, nail, plaster of paris) (p = 0.395). The time of treatment onset didn’t affect the incidence of fracture healing (p = 0.841). Although statistical significance was not demonstrated, longer treatment duration showed a trend of increased probability of union (p = 0.081).

**Conclusion:**

PEMF stimulation is an effective non-invasive method for addressing non-infected tibial union abnormalities. Its success is not associated with specific fracture or patient related variables and it couldn’t be clearly considered a time-dependent phenomenon.

## Background

It has been traditionally quoted that 5-10% of fractures worldwide may develop delayed union or nonunion [1]. Considering that in United States alone the number of fractures is 7.9 million annually, it is widely accepted that fracture union abnormalities have a significant clinical and financial impact on health care systems [1]. Surgical management with debridement of necrotic tissue and rigid fixation (either internal or external) along with a form of biological enhancement, such as bone grafting, is considered the ‘gold standard’ for the treatment of nonunions [2]. However, non-invasive treatment options including low-intensity pulsed ultrasound (LIPUS), extra-corporeal shock wave therapy, electrical and pulsed electromagnetic fields (PEMF) stimulation have been also suggested for the management of nonunited fractures [3].

We present the results of PEMF stimulation in treating non-infected tibial delayed unions and nonunions. Factors that might affect the success of the method were investigated and a thorough literature review was also conducted to assess the overall efficacy of the method in tibial nonunited fractures.

## Methods

**Figure 1:**
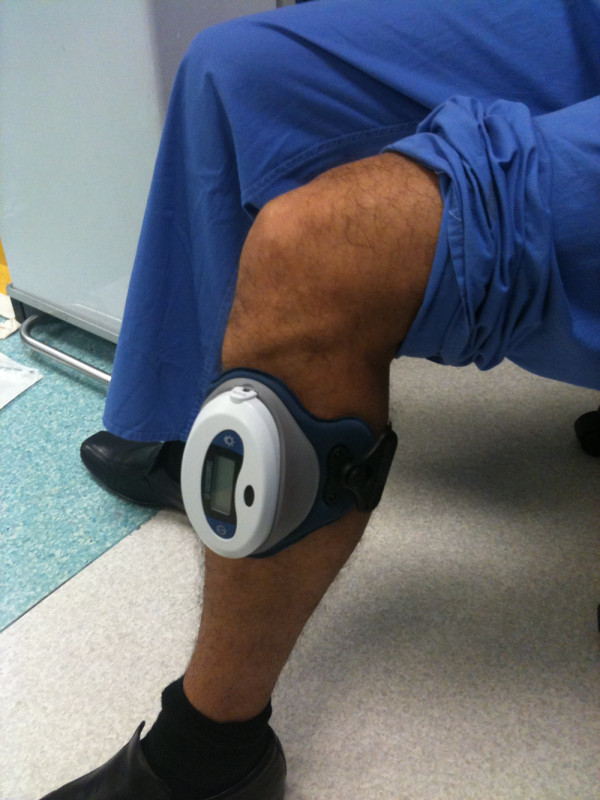
PEMF device (Physio-Stim, Orthofix).

We prospectively evaluated 52 consecutive patients with tibial shaft delayed union (and nonunion who were treated with a battery-powered PEMF device (Physio-Stim, Orthofix) (Figure1). Eight patients were excluded from the study due to lost to follow-up (5 patients), non-attendance the outpatient appointments (2 patients) and severe vascular dementia (1 patient) leaving 44 patients for further evaluation. The medical records of these patients were assessed with the approval of the hospital's institutional review board.

In accordance with the manufacturer guidelines, the system was applied 3 hours per day, for a maximum period of 36 weeks. Further weight-bearing restrictions or fracture immobilization were not applied. The fracture gap in all cases was less than 1 cm and the bone defect less than one-half the width of the bone to be treated. Patients with infective nonunion or severe soft-tissue defect were not deemed candidates for the PEMF stimulation regime.

The device was prescribed by the treating orthopaedic surgeon during the outpatient clinic reviews. The prescription was then reviewed by the Clinical Director of the department and was supplied by the senior plaster technician, who was in direct communication with the manufacturer. Patients were monthly followed-up using serial anteroposterior and lateral X-Rays until fracture union occurred or further operation took place. The plain radiographs were reviewed in order to assess the initial fracture type and progress of fracture healing. The absence of either adequate fracture callus in a minimum period of 9 months or progression toward healing for 3 consecutive months was defined as nonunion. Delayed union was assumed when no union was achieved at 20–26 weeks postoperatively. The fracture considered to be healed when radiographic evidence of bridging callus formation was seen in at least three cortexes.

## Statistics

Statistical analysis was performed with the use of SPSS 17. Variables were tested using normality plots and the Kolmogorov-Smirnov test (with 0.200 considered as the lower boundary of true significance). Non-parametric numerical variables are presented as median, with range between round brackets and were compared with the Mann–Whitney U test. Normally distributed numerical data are presented as mean with standard deviation (SD is symbolized with ±) in brackets and were compared using the student’s t-test. The Chi-square test was used to study categorical variables. Correlation between scale variables was analyzed with Spearman’s rho. Kaplan-Meier survivorship curve was used for analysis of the probability of fracture union. Statistical significance was assumed at p < 0.05.

## Results

### Demographics

There were 17 women and 27 men with a mean age of 49.6 ± 18.4 years. The left limb was affected in 25 patients and the right in 19 patients. Fifteen out of the 44 patients were smokers. Diabetes was present in 5 patients.

From a total of 44 fractures, 17 were simple closed fractures, 10 were comminuted closed fractures, 8 were Grade I open fractures, 3 were Grade II open fractures, 4 Grade IIIA open fractures and 2 Grade IIIB open fractures (according to Gustilo-Anderson classification [4]). The fractures were initially treated with external fixator (6 cases), intramedullary nail (12 cases), plating without bone grafting (10 cases), plate with bone-grafting (7 cases), or Plaster of Paris (POP) (9 cases).

### Union

**Figure 2:**
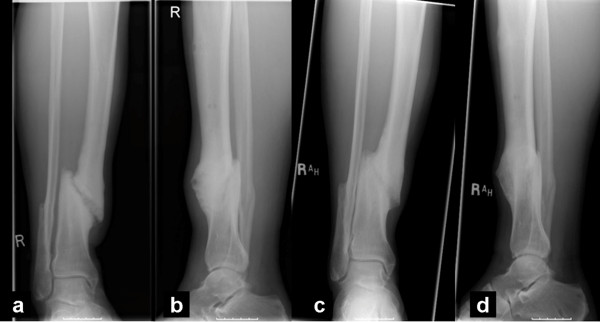
**Anteroposterior (a) and lateral radiographs (b) of a distal tibial nonunion 10 months after fracture.** PEMF stimulation of fracture site led to fracture union 5 months thereafter as shown in anteroposterior ( **c**) and lateral ( **d**) tibial views.

**Figure 3:**
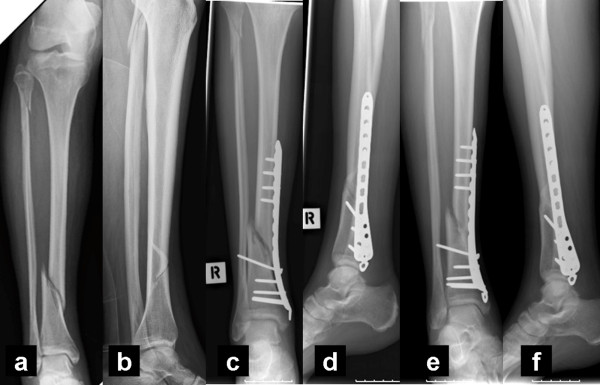
**Anteroposterior (a) and lateral radiographs (b) of an open Grade I distal tibial fracture.** Six months after surgery, anteroposterior ( **c**) and lateral tibial radiographs ( **d**) showed slow progression of healing. Seven months after PEMF introduction, anteroposterior ( **e**) and lateral tibial radiographs ( **f**) showed bridging callus in 3 out of 4 cortexes (medial, anterior and posterior).

Fracture union was achieved in 34 out of 44 cases (77.3%) (Figures 2 &3). The 10 nonunions were observed in 2 simple closed fractures (1 smoker), in 3 comminuted closed fractures (one smoker), in 2 Grade I open fractures (one smoker), in 2 Grade IIIA open fractures (one smoker) and in one Grade IIIB open fracture (one diabetic). No statistical significant relationship was found between union rate and age (t-test, p = 0.819), smoking status (chi-square test, p = 0.245), diabetes (chi-square test, p = 0.681), (fracture side (left or right) (chi-square test, p = 0.734), fracture type (simple or comminuted, open or closed) (chi-square test, p = 0.111) and initial treatment method applied (p = 0.395, chi-square test).

**Figure 4:**
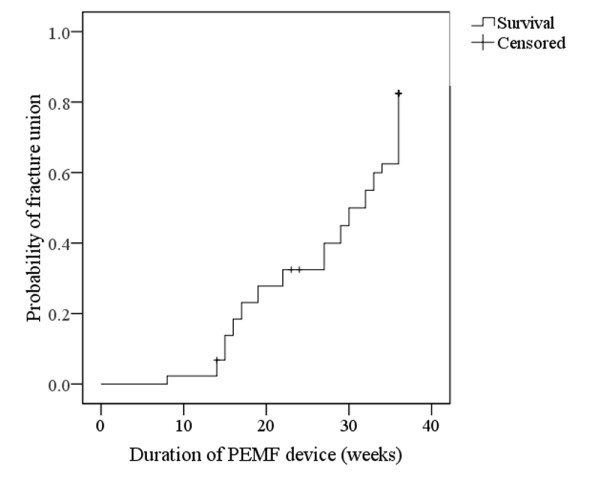
**Kaplan-Meier recurrence curve of patients treated with PEMF.** Line represents probability of fracture union in relation to the duration of PEMF treatment (weeks). Crosses represent censored cases where follow-up was stopped because union was deemed unlikely with conservative management.

The median time interval between the latest fracture management and the introduction of PEMF was 24.5 weeks (21–57 weeks)**.** The median duration of PEMF application was 29.5 weeks (8–36 weeks). No correlation was observed between these two time variables (p = 0.348, r = 0.145, spearman rho test). Similarly, no association was found between the above time variables and the overall union rate (p = 0.081 and p = 0.841, respectively, Mann Whitney U test). The probability of fracture union in relation to duration of PEMF is presented in Figure4. Although statistical significance was not demonstrated, the curve shows a trend of increased probability of healing after longer application of the device.

## Discussion

The use of electrical stimulation in fracture healing is not a novel concept. There have been relevant reports from as early as 1841 [1] but the use of this method did not become widespread until the early 1950s, when Yasuda [5] demonstrated new bone formation in rabbit femora, adjacent to a cathode. He also demonstrated that there were electric potentials in bones, that were categorized into steady-state and stress-induced potentials [5]. The latter develop when a bone is subjected to a bending force, which causes the compressed side to become negatively charged when compared to the tensed side of the bone [6]. Steady-state potentials are potentials that arise in areas of bone activity and are independent to stresses.

Until the late 1970s there was an abundance in the literature of reports describing the effects of electricity on bone growth and fracture repair [1]. Since then, a variety of devices have been developed in order to produce electromagnetic fields to the fracture site. Recent and more widespread PEMF devices utilize non-invasive inductive coupling and can be used along with every method of fracture fixation [7]. Interestingly, the electrical stimulation market is approximately worth 500 million dollars in the United States [8].

It seems that the introduction of electromagnetic fields at the fracture site can stimulate the bone in a way similar to mechanical loading [1]. However, there is still ongoing debate regarding the mechanism of action of PEMF at cellular and molecular level. PEMF have been advocated to stimulate the synthesis of extracellular matrix proteins and exert a direct effect on the production of proteins that regulate gene transcription [9]. Electromagnetic fields may also affect several membrane receptors including PTH, insulin, IGF-2, LDL and calcitonin receptors [10]. Moreover, when osteoblasts are stimulated by PEMF, they secrete several growth factors such as bone morphogenic proteins 2 and 4 and TGF-beta [6,11].

The principle underlying the application of PEMF is that of inductive coupling [6,11]. The electric current is produced by a coil, driven by an external field generator. The outcome is a secondary electrical field produced in the bone [1]. The secondary field is dependent on the characteristics of the applied magnetic field and tissue properties. Magnetic fields varying from 0.1 to 20 G are usually applied in order to produce electrical fields in bone, ranging from 1 to 100 mV/cm [11]. Contra-indications to the use of PEMF include segmental bone loss, infected nonunions, synovial pseudarthrosis and poor stability of fracture site [11].

As opposed to other methods of non-invasive augmentation of fracture healing, such as low-intensity pulsed ultrasound (LIPUS), PEMF have not been assessed thoroughly in robust studies of high methodological quality [1]. Despite the relative scarcity of well-organized randomized controlled trials, many in vivo and in vitro studies highlight the method’s potential usefulness [11]. Particularly and in terms of clinical practice, the efficacy quoted in treating tibial delayed unions or nonunions has been reported to range between 45% and 87% [12-20] (Table1).

**Table 1:** Summary of clinical studies using Pulsed Electromagnetic Field therapy in tibial delayed unions and nonunions

***Study***	***Year***	***Design***	***Number of tibia fractures***	***Mean treatment duration***	***Hours per day***	***Union rate***
**De Haas (17)**	1980	Prospective, non-randomized	17	23.6 weeks	20	88.2%
**Bassett (15)**	1981	Prospective, non-randomized	127	5.2 months	10	87%
**Sharrard (19)**	1982	Prospective, non-randomized	30	6 months	12 to 16	86.7%
**Barker (14)**	1984	Prospective, randomized, double-blind	16	24 weeks	12 to 16	55.6%
**De Haas (16)**	1986	Prospective, non-randomised	56	ND	ND	84%
**Sharrard (18)**	1990	Prospective, randomized, double-blind	45	12 weeks	12	45%
**Scott (22)**	1994	Prospective, randomized, double-blind	15	26.8 weeks	ND	60%
**Simonis (20)**	2003	Prospective randomized, double-blind	34	6 months	ND	70.6%
**Gupta (21)**	2009	Prospective, non-randomized	45	8.35 weeks	ND	85%
**Current study**	2011	Prospective, non-randomized	44	29.5 weeks	3	77.3%

ND: Not Defined.

One of the first series that examined the results of PEMF treatment on delayed unions was published in 1980 by De Haas et al [15]. Their series comprised of 17 patients with tibial fracture abnormalities and the reported healing rate was 88.2%. Despite the small sample size and lack of randomization and blinding, this study was considered significant, as it was the first that demonstrated the potential benefit of PEMF in promoting fracture healing.

Bassett et al [13] reported a case series of 127 nonunited or delayed united tibial fractures that treated with PEMF. Patients were recruited over a 5-year period after an average of 2.4 failed surgical interventions prior to PEMF application. PEMF were applied for a mean period of 5.2 months and the patients were advised to remain non-weight bearing. The overall healing rate was 87%. Sharrard et al [17] found a 86.7% successful outcome in 53 tibial nonunions that treated with a PEMF system. The authors advocated that infection, a screw in the fracture gap, a gap of more than 5 mm and inadequate immobilization were responsible for treatment failure. Gupta et al [19] studied prospectively 45 tibial atrophic nonunions without infection, presence of implants or fracture gap more than 1 cm. Healing was achieved in 85% of cases during a 4-month period. Poor compliance was considered responsible for the three persistent nonunions.

A randomized double-blind clinical trial examining 34 tibial nonunions demonstrated a statistically significant increase in union rate when a PEMF device was administered [18]. However, a recent meta-analysis, which pooled the data from eleven studies, showed that PEMF resulted in a non-significant increase of healing rate of long bone nonunions or delayed unions [8]. A potential problem with the meta-analysis was that the included studies used different settings of PEMF and they set different endpoints. The authors concluded that the available clinical evidence was insufficient to conclusively suggest a clinical benefit of this method in the management of bone nonunions [8]. It seems that more randomized controlled trials with a high number of patients are necessary to clarify the overall cost-effectiveness of the method for the treatment of tibial or long-bone delayed unions and nonunions.

In our study, the healing rate was 77.3% but patient or fracture variables as well as time of treatment onset didn’t affect the healing rate. Longer periods of PEMF application were associated with a trend for increased union probability but no statistical significance was achieved. However, the study contained some important limitations. These were its non-randomized nature, the small sample size and the fact that certain crucial data, such as the use of non-steroidal anti-inflammatory medications, weren’t taken into consideration.

## Competing interests

The author(s) declare there are no competing interests.

## Authors’ contributions

AA reviewed the patients clinically and radiologically, collected the data and reviewed the literature. NS statistically analyzed the data and wrote the first draft of the paper. BC conceived, designed and revised the study. Each author read and approved the final manuscript.
